# The Psychological Impact of Amblyopia Treatment: A Systematic Literature Review

**DOI:** 10.22599/bioj.426

**Published:** 2025-01-16

**Authors:** Louisa Haine, Isaac Taylor, Megan Vaughan

**Affiliations:** 1 Anglia Ruskin University, UK; 2 UCL, UK

**Keywords:** amblyopia, treatment, occlusion, psychological distress, review

## Abstract

**Aim:**

The aim of this literature review was to determine if a consensus could be reached on whether amblyopia treatment causes distress to patients and/or their guardians, and if so, establish the impact of this reported psychological distress upon paediatric patients and/or their parents/guardians.

**Methods:**

A systematic review of the literature was conducted of all publications written in English. Search terms included both MeSH terms and alternatives related to amblyopia and psychological distress. Evidence quality was assessed using an adapted Newcastle-Ottawa Score (NOS) and evaluation of the literature was used to form a narrative synthesis of the findings.

**Results:**

Initial searches yielded 7,838 titles in total, with 25 peer reviewed papers published between 1999 and 2021 meeting the study inclusion criteria. Factors such as the presence of strabismus, moderate and severe amblyopic density, occlusive patch treatment and patching during school age increase the likelihood of experiencing distress as a result of amblyopia treatment.

**Conclusions:**

Both parents/guardians and patients can experience psychological distress as a result of undertaking amblyopia treatment. School-aged children and those receiving occlusion therapy in the form of patching report higher distress than infants and young-children, and those receiving atropine occlusion therapy or refractive correction only. Further study measuring the physiological markers of distress such as Cortisol and BDNF, is recommended.

## 1. Introduction

Amblyopia is a predominantly unilateral, visual neurodevelopmental disorder, contingent on the absence of any ocular pathology. With an estimated prevalence of around 3% in British children under 15 years, amblyopia is the most common cause of visual impairment in childhood (Thompson *et al*., 1991). Amblyogenic factors such as the presence of strabismus, anisometropia or a stimulus deprivation occurring within the visual development critical period (Daw, 1998), instigate abnormal development of the visual cortex (V1), resulting in poor visual acuity, as well as impaired contrast sensitivity and stereopsis. If untreated during childhood, these visual deficits become permanent. Throughout the lifetime, the presence of amblyopia may hinder a child’s educational and social development (Packwood *et al*., 1999; Satterfield, Keltner and Morrison, 1993), limit future career opportunities (Carlton and Kaltenthaler, 2011), and almost doubles the lifetime risk of bilateral visual impairment (Rahi *et al*., 2000; van Leeuwen *et al*., 2007).

Due to physiological reductions in neuroplasticity throughout childhood, it is considered that effectiveness of amblyopia treatment is substantially diminished beyond the age of eight-years, therefore earlier commencement of treatment is associated with better visual outcomes (Chen and Cotter, 2016). Due to the time-limited nature of the effectiveness of amblyopia treatment, paediatric visual screening programmes are employed to identify individuals with amblyopia.

Current clinical treatment options for amblyopia include refractive correction (where required), followed by occlusion therapy, such as patching and/or optical penalisation, such as with Atropine (1%) to temporarily deprive the non-amblyopic eye of clear vision (Chen and Cotter, 2016). While patching treatment tends to be utilised more frequently, treatment efficacy has been shown to be similar for both patching and atropine modalities in cases of moderate amblyopia in children aged 5–7 years, with atropine scoring slightly higher (better) in parental measures of acceptability (The Pediatric Eye Disease Investigator Group, 2002). The British and Irish Orthoptic Society ‘Overview of Amblyopia Practise’ recommends that, where appropriate, parents should be offered a choice of treatment between occlusion therapy or atropine (British and Irish Orthoptic Society, 2016).

The success of amblyopia treatment is contingent on factors such as amblyopia type, amblyopia density, duration of occlusion and treatment compliance (Fielder *et al*., 1994; Fielder *et al*., 1995; Newsham, 2000; Newsham, 2002; Smith *et al*., 1995; Woodruff *et al*., 1994). While treatment compliance is a critical factor for the success of amblyopia treatment (Lithander and Sjöstrand, 1991), non-compliance rates with treatment have reported to be as high as 54% (Newsham, 2000), with commonly cited reasons for non-compliance being psychosocial in nature (Wang, 2015). However, while some studies report that amblyopia treatment is generally well accepted by both the child and their parents (Choong *et al*., 2004; Holmes *et al*., 2003; Hrisos, Clarke and Wright, 2004) with no long-term emotional or behavioural disturbance seen in children who received amblyopia treatment (Smith *et al*., 1991), others report children experiencing significant feelings of shame, embarrassment (Koklanis, Abel and Aroni, 2006) as well as an increased likelihood of verbal or physical bullying from their peers (Horwood *et al*., 2005). Furthermore, it is shown that higher levels of distress experienced by parents impacts not only upon treatment compliance (Eddy *et al*., 1998; Otero and Hodes, 2000) and consequently outcome success but may also impact upon their children’s behavioural and emotional state if distress levels intensify significantly leading to dysfunctional parenting (Hadadian and Merbler, 1996). It is therefore important to consider the psychosocial impact of amblyopia treatment to the patient and their family, both during the treatment process and beyond.

For this reason, we conducted a systematic literature review to evaluate and summarise studies that examined the psychological impact/effect of amblyopia treatment on patients and their guardians to establish whether a consensus could be reached on whether amblyopia treatment causes distress to patients and/or their guardians. Furthermore, the review aimed to consider the potential impact of any amblyopia treatment-initiated distress upon the lives of patients and/or their guardians, and where possible to identify any key modifiable factors that may help mitigate for any negative impact seen.

## 2. Methods

A systematic review was conducted to form a narrative synthesis and analysis of the findings. The review both observed and is reported according to PRISMA guidelines (Appendix 1), and was registered with the International Prospective Register of Systematic Reviews (PROSPERO) (CRD42023434054). PROSPERO is a recognised international database that was launched in 2011 to increase transparency of systematic reviews (Booth *et al*., 2011).

### 2.1 Inclusion criteria

#### 2.1.1 Study types

The following types of studies were included in this systematic literature review: primary observational studies (including cross-sectional studies and prospective cohort studies), randomised and non-randomised controlled trials, and case-controlled studies. Individual case reports, editorials, letters, and any other articles deemed not relevant to this review were excluded. All studies were required to be published in English.

#### 2.1.2 Participant types

We included studies of human participants with amblyopia or those with amblyogenic factors, who were undergoing or had undergone amblyopia treatment, and/or their guardians. Studies which included participants with non-amblyopia co-morbidities were included if the data and findings relevant to the amblyopic participants could be isolated and extracted.

#### 2.1.3 Outcome and data types

Data types included for consideration as part of the narrative synthesis included patient or parent led questionnaires which contained an evaluation of distress or worry, or structured interviews leading to thematic analysis which included discussion of psychological and social impacts of amblyopia.

### 2.2 Amblyopia definition

Amblyopia is defined here as reduced unilateral or bilateral visual acuity, not immediately correctable by refractive correction, and present in the absence of ocular pathology (Powell *et al*., 2005; Stewart *et al*., 2002).

### 2.3 Search methods for literature identification

Systematic search strategies were employed to search electronic databases (MEDLINE, EMBASE, CIHAHL, PsychINFO, Web of Science and Scopus, date searches conducted: 14/06/2023). Search terms used can be seen in Appendix 2. Additionally, manual reference searches also occurred, searching the reference lists of all included studies. Search terms included both controlled ‘Medical Subject Heading’ (MeSH) terms and alternatives related to amblyopia and psychological distress (Appendix 2).

### 2.4 Study selection

Following the preliminary systematic literature search and removal of duplicates, all titles and abstracts were independently screened for inclusion by two review authors (LH and IT). Studies meeting inclusion criteria were then reviewed in full independently by the two review authors (LH and IT) before making a final decision on eligibility. The reference lists of included studies were manually searched for references which met the title and abstract inclusion criteria, and these were again reviewed in full independently by the two review authors (LH and IT) before making a final decision on eligibility. In cases of discrepancies between the review authors regarding study eligibility, the opinion of a third review author (MV) could be called upon, where necessary.

### 2.5 Data extraction

A pre-designed data extraction form was used to gather information from included studies on authors, aim/objective, date of study, country, study type, single vs. multicentre, method of amblyopia therapy, method of evaluation of distress, number of participants, type of participants (patient and/or guardian), duration of observation/evaluation, outcomes, comparator, inclusion and exclusion criteria, conflicts of interests, limitations, and conclusions. Data was extracted by one review author (IT) and verified by a second review author (LH).

### 2.6 Quality assessment

Risk of bias was assessed by two independent researchers (LH and IT) with the aid of an adapted version of the Newcastle-Ottawa (Scale (NOS) checklist (Tramontano *et al*., 2021; Wells *et al*., 2000). This NOS is a star-based system which is categorised into three groups: selection of the study groups, the treatment protocol, and outcome(s). Stars are awarded for each quality item with the highest quality studies having up to seven stars (2 stars for the selection group, 2 stars for the treatment protocol group, and 3 stars for the outcome(s) group) (Appendix 3). Discrepancies between the review authors over the risk of bias in particular studies were made by consensus, with involvement of the third review author (MV) where necessary, which occurred only once during this study. The overall methodological quality was high, ranging from a minimum of 4 stars, to a maximum of 7 stars (Appendix 3).

### 2.7 Data analysis

Considering the heterogeneous nature of identification and measurement of psychological distress, a narrative synthesis and analysis of the findings was undertaken. This narrative analysis was structured around the type of evaluation, including a report of methodological approaches and outcome measures as reported in the publications (or acquired from the authors, where applicable). Descriptive statistics such as study population characteristics (both personal and clinical) were extracted to support the narrative analysis, where available and appropriate.

## 3. Results

**Figure 1:**
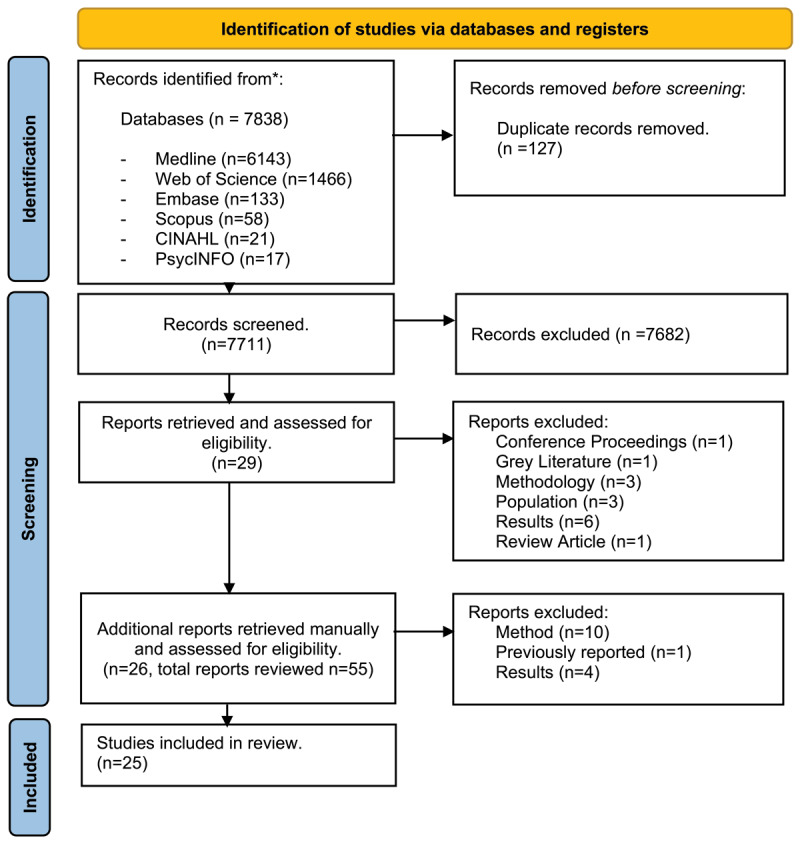
Flowchart of search procedures used.

The literature search yielded a total of 7,838 articles. Following the removal of 127 duplicate articles, 7,711 underwent title and abstract screening. A total of 55 full-text articles were retrieved and assessed for eligibility. Finally, 25 peer review papers, published between 1999 and 2021, were found to meet the study inclusion criteria (Figure 1).

### 3.1 Study characteristics

Characteristics of all included studies can be found in Table 1.

**Table 1:** Characteristics of included studies.


FIRST AUTHOR	YEAR	TOTAL (n)	COUNTRY	SINGLE OR MULTICENTRE	METHODOLOGY	METHOD OF EVALUATION OF DISTRESS	PERSONS EXAMINED			NOS QUALITY SCORE

							PATIENT PERSPECTIVE	GUARDIANS’ PERSPECTIVE OF THEMSELVES	GUARDIANS’ PERSPECTIVE OF PATIENT	

Alkulaib	2021	100	Saudi Arabia	Multicentre	Quantitative	Perceived stress scale (PSS)		✓		7

Bhandari	2012	52*	Nepal	Single	Mixed methods	Semi-structured interviews and questionnaires (parental questionnaire and emotional impact questionnaire)	✓	✓	✓	7

Carlton	2013	59	UK	Multicentre	Mixed methods	Semi-structured interviews and Child Amblyopia Treatment Questionnaire (CAT-QoL)	✓			6

Chen	2016	44	China	Single	Quantitative	Health Related Quality of Life questionnaire (HRQoL)	✓			7

Choong	2004	65	UK	Single	Quantitative	Study specific carer’s questionnaire in 5 sections; Background information on the carer and child, Carers knowledge on amblyopia and compliance to treatment, Perceived Stress Index (PSI), Perceived Psychosocial Questionnaire (PPQ), Carers relationship with the child and other family members.		✓	✓	7

Cole	2001	64	USA	Multicentre	Quantitative	Amblyopia Treatment Index (ATI)		✓	✓	7

Dixon-Woods	2006	25	UK	Single	Qualitative	Semi-structured interviews		✓	✓	5

Drews	2003	41	USA	Multicentre	Quantitative	Parenting Stress Index (PaSI), Ocular Treatment Index (OTI)		✓		7

Drews-Botsch	2019	111	USA	Multicentre	Quantitative	Parenting Stress index (PaSI)		✓		6

Drews-Botsch	2012	104	USA	Multicentre	Quantitative	Parenting Stress Index (PaSI), Ocular Treatment Index (OTI)		✓		7

Felius	2010	378	USA	Multicentre	Quantitative	Parent and Child Amblyopia Treatment Index (ATI)	✓	✓	✓	7

Guimarães	2019	79	Portugal	Single	Quantitative	KIDSCREEN-52 (K52), Depression, Anxiety and Stress Scale for Children (EADS-C), Beck Anxiety Inventory (BAI), Hospital Anxiety and Depression scale (HADS), World-health Organization Quality of Life instrument (WHO-QOL-BREF), NEO Five-Factor Inventory (NEO-FFI), 10-item Perceived Psychological Scale (PSS-10)	✓	✓		6

Holmes	2003	419	USA	Multicentre	Quantitative	Amblyopia Treatment Index (ATI)		✓	✓	7

Holmes	2008	794	USA	Multicentre	Quantitative	Amblyopia Treatment Index (ATI)		✓	✓	7

Hrisos	2004	144	UK	Multicentre	Quantitative	A study specific questionnaire – Emotional Impact Questionnaire correlated against the Revised Rutter Scale for Preschool children.		✓	✓	7

Kitasato	2020	98	Japan	Multicentre	Quantitative	Kessler Psychological Distress Scale (K6), study-specific questionnaire examining status and emotions.		✓	✓	6

Koklanis	2006	91	Australia	Single	Quantitative	The Behaviour Assessment System for Children Parent rating scale (BASC-PRS) and the Behaviour Assessment System for Children Self Report Scale (BASC-SRP)	✓	✓	✓	6

Loudon	2009	149	The Netherlands	Multicentre	Mixed methods	A study specific questionnaire based on protection motivation theory (PMT) known as the Patching Success Questionnaire (PSQ), and semi-structured interviews for non-compliant patients		✓	✓	7

Packwood	1999	25	USA	Multicentre	Quantitative	A Study-specific questionnaire using 5-point rating scale and The Hopkins Symptoms Checklist (HSC)	✓			6

Parkes	2001	59	UK	Single	Quantitative	A study specific questionnaire			✓	7

Sabri	2006	240	UK	Multicentre	Quantitative	The Visual Function (VF14) and an 8-item study specific psychological questionnaire	✓			6

Searle	2002	151	UK	Multicentre	Quantitative	A study specific questionnaire based on protection motivation theory (PMT)		✓	✓	6

Searle	2000	20	UK	Single	Qualitative	Semi-structured interviews based on protection motivation theory (PMT)		✓	✓	4

Tjiam	2011	52	The Netherlands	Multicentre	Mixed methods	Structured oral questionnaire based on the ‘Social Position and Use of Social Services by Migrants and Natives’ Questionnaire (SPVA), with a study specific additional domain of ‘Lazy Eye’ comprising of an additional 33 questions.		✓	✓	6

Webber	2008	99	Australia	Single	Quantitative	SPPC	✓			7


* Guardian numbers not reported.

### 3.2 Psychological assessment

Methods of psychological examination were varied. The Amblyopia Treatment Index (ATI) (Cole *et al*., 2001; Felius *et al*., 2010; Holmes *et al*., 2003; Holmes *et al*., 2008) and the Child Amblyopia Treatment Questionnaire (CAT-QoL, Carlton, 2013) were the only amblyopia treatment-specific questionnaires identified. The ATI, developed by the Paediatric Eye Disease Investigator Group (PEDIG), is an 18-item questionnaire designed to quantify the impact of amblyopia treatment for both the patient and their guardian/family (Felius *et al*., 2010; Holmes *et al*., 2003; Holmes *et al*., 2008), while during the development of the CAT-QoL, 11 key themes were identified which included physical sensation of treatment, social impact, physical ability and emotions towards self and the family unit (Carlton, 2013; See Appendix 4 for details).

The majority of research used more generalised, non-amblyopia specific, forms of assessment such as:

- Semi-structured interviews (Bhandari, Sharma and Shrestha, 2012; Carlton, 2013; Dixon-Woods, Awan and Gottlob, 2006; Kitasato *et al*., 2020; Koklanis, Abel and Aroni, 2006; Loudon *et al*., 2009; Searle *et al*., 2000)
- Study specific-designed questionnaires (Bhandari, Sharma and Shrestha, 2012; Choong *et al*., 2004; Hrisos, Clarke and Wright, 2004; Kitasato *et al*., 2020; Packwood *et al*., 1999; Parkes, 2001; Sabri *et al*., 2006)
- Study specific-designed questionnaires based on protection motivation theory (PMT – the theory that people are more likely to protect themselves from a health threat when they perceive the threat as significant, believe they are likely to experience negative consequences, and believe they can effectively reduce the threat) (Loudon *et al*., 2009; Searle *et al*., 2002)
- Health-Related Quality of Life (HRQOL) questionnaires (Chen *et al*., 2016) including the paediatric KIDSCREEN-52 (K52) (Guimarães *et al*., 2019) and the World Health Organization Quality of Life instrument (WHO-QOL-BREF) (Guimarães *et al*., 2019)

***Further psychological assessments included:***

- The Hopkins Symptom Checklist (HSC) (Packwood *et al*., 1999)
- The Behaviour Assessment System for Children Parent rating scale (BASC-PRS) and the Behaviour Assessment System for Children Self Report Scale (BASC-SRP) (Koklanis, Abel and Aroni, 2006)
- The Harter Self Perception Profile for Children (SPPC) (Webber *et al*., 2008)
- The Depression, Anxiety and Stress Scale – Children (EADS-C) (Guimarães *et al*., 2019)
- The Beck Anxiety Inventory (BAI) (Guimarães *et al*., 2019)
- The Perceived Stress Index (PSI) (Choong *et al*., 2004)
- The Perceived Stress Scale (PSS) (Alkulaib *et al*., 2021)
- The 10-item Perceived Psychological Scale (PSS-10) (Guimarães *et al*., 2019)
- The Hospital Anxiety and Depression Scale (HADS) (Guimarães *et al*., 2019)
- The NEO Five-Factor Inventory (NEO-FFI) (Guimarães *et al*., 2019)
- The Parenting Stress Index (PaSI) (Drews *et al*., 2003; Drews-Botsch *et al*., 2012; Drews-Botsch *et al*., 2019)
- The Ocular Treatment Index (OTI) (Drews *et al*., 2003; Drews-Botsch *et al*., 2012)
- The Perceived Psychological Questionnaire (PPQ) (Choong *et al*., 2004)
- The Kessler Psychological Distress Scale (K6) (Kitasato *et al*., 2020)
- The Visual Function 14 (VF-14) (Sabri *et al*., 2006)
- The ‘Sociale Positie en Voorzieningenge-bruik Allochtonen en Autochtonen’ (SPVA, meaning ‘Social Position & Use of Social Services by Migrants and Natives’; Tjiam *et al*., 2011)

**Figure 2:**
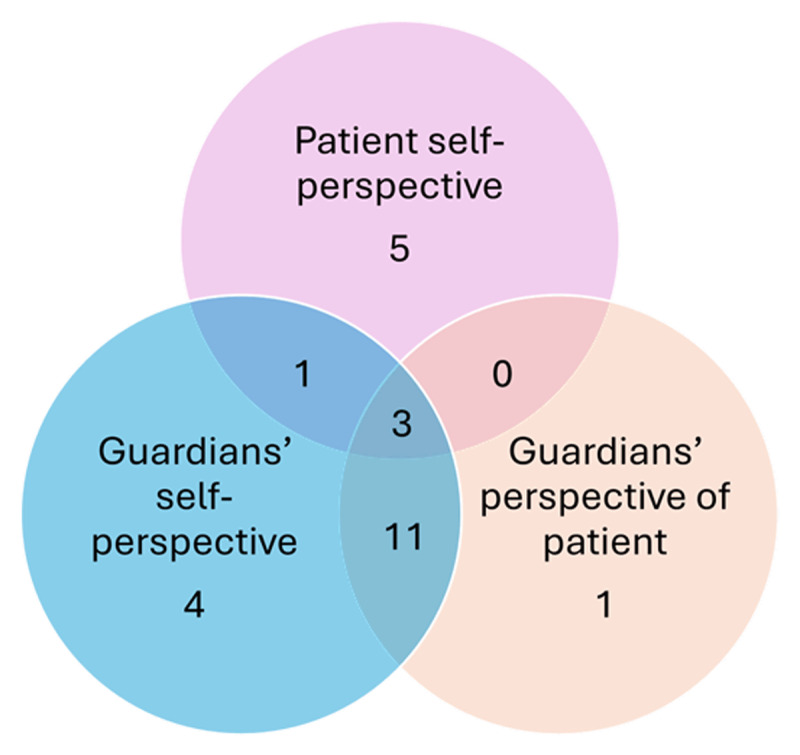
Venn diagram demonstrating the groups and perspectives assessed, and number of studies that examined them.

The majority of the identified studies (16/25, 64%) examined only the parental perceptions of the amblyopia treatment process. In total, only nine (36%) of the studies examined the amblyopic patient’s perspective directly, and only 4/25 (16%) examined and considered both parental and patient perspectives (Figure 2).

Of the 25 studies identified, 23 (92%) demonstrated a perceived increase of the presence of distress in at least some of their participants, however this did not always reach statistical significance (where statistical analysis was completed). The proportion of individuals affected and extent to which participants were impacted psychologically by amblyopia treatment, varied considerably between these studies. Of the five studies where patients perceptions were directly considered (Bhandari, Sharma and Shrestha, 2012; Felius *et al*., 2010; Guimarães *et al*., 2019; Koklanis, Abel and Aroni, 2006) only one concluded that the patients were not impacted psychologically by their amblyopia treatment, however this was a retrospective study and so was only able to conclude about the long-term psychological impact (≥1 year post treatment cessation; Guimarães *et al*., 2019). Several key themes were identified throughout the review which were considered to influence the presence of perceived distress such as patient age, amblyogenic factor, amblyopia severity, treatment modality and duration, treatment compliance and stigmatisation. These themes are explored further below.

### 3.3 Patient age

Parental perceptions generally regarded that younger children (pre-school age) were able to cope better with amblyopia treatment and demonstrated better compliance than their school aged counterparts (Searle *et al*., 2000); additionally, pre-school children undergoing amblyopia treatment were considered happy and sociable in childcare settings (Hrisos, Clarke and Wright, 2004).

Negative responses to patching are reported to appear around school age (Koklanis, Abel and Aroni, 2006), with 90% of Nepalese parents expressing that patching upset their child, but these negative responses were only reported in children aged >5 years (Bhandari, Sharma and Shrestha, 2012). These difficulties may occur as children begin to formally socialise with their peers at school, with the presence of an occlusive patch leading to feelings of self-consciousness, embarrassment, and shame (Koklanis, Abel and Aroni, 2006; Webber *et al*., 2008). Concerns surrounding self-perception can also lead to poorer treatment compliance (Searle *et al*., 2000).

Finally, in comparisons between age groups, studies reported no significant difference was seen in social acceptance scores between patching at pre-school age and patching at school age (p = 0.339) (Webber *et al*., 2008), and amblyopia treatment index (ATI) scores achieved in 7 to <13-year-olds have been shown to be similar to those of 3 to <7-year-olds (Felius *et al*., 2010).

Retrospectively, reports from adults who had received amblyopia treatment as a child, demonstrated that up to 21% of patients exhibited concern regarding the effect that amblyopia and its treatment had had upon their childhood self-image, with treated amblyopes displaying a significantly greater degree of somatization, obsession-compulsion, inter-personal sensitivity, depression, and anxiety compared with control groups (p < 0.001) (Packwood *et al*., 1999). Additionally, adults who had undergone amblyopia treatment as a child and found occlusion to be an unpleasant experience, yielded significantly larger detrimental psychological impact scores than those who found occlusion to be an acceptable experience (p < 0.0001) (Sabri *et al*., 2006).

In consideration of parental distress, the literature supports the idea that implementation of occlusion therapy (patching) does not significantly increase levels of stress experienced in parents of pre-school aged amblyopes, as demonstrated by studies of children undergoing occlusion therapy for treatment of unilateral congenital cataracts (UCC) compared to pre-school aged children with bilateral congenital cataracts (BCC) (Drews *et al*., 2003; Drews-Botsch *et al*., 2019). Among parents of patients under one year of age taking part in the Infant Aphakia Treatment Study, only 10% of parents reported above average personal stress levels (Drews-Botsch *et al*., 2012). Beyond infancy (>12 months) studies have detected increased parental worry and distress in relation to amblyopia treatment (Bhandari, Sharma and Shrestha, 2012), particularly once children with amblyopia reach school-age (4–5 years of age) with 58–62% of guardians expressing worry about amblyopia treatment and 39–62% of guardians specifically expressing distress instigated by their child’s treatment (Hrisos, Clarke and Wright, 2004). In a more recent study of parents/guardians of Saudi Arabian amblyopic children (Alkulaib *et al*., 2021), overall perceived parental stress levels were reported as ‘moderate’ to ‘high’ in 90% of parents, with these higher stress levels being most apparent when the patients were 6–10 years of age, although this difference between patient age groups did not reach statistical significance (p = 0.120).

### 3.4 Stigmatization, peer victimisation and compliance

Bullying and teasing were documented as common experiences for children undergoing amblyopia treatment, particularly in older children (>7 year of age; Bhandari, Sharma and Shrestha, 2012) and were commonly associated with spectacle and/or occlusive patch wear (Koklanis, Abel and Aroni, 2006), along with the presence of strabismus (Sabri *et al*., 2006). Using the Harter Self Perception Profile for Children, children with amblyopia have also been shown to score significantly lower in domains of social acceptance than age-matched control children (p = 0.012), with significantly poorer scores seen in amblyopic children who had received patching occlusion therapy than those who did not (p = 0.024) (Webber *et al*., 2008). Consequently, children displayed less comfort discussing their treatment among peers than with their adult family members, with some children exhibiting secretive behaviours in an attempt to hide their treatment from their peers. In addition to a high degree of distress, increased perceived stigmatisation was also associated with poorer treatment compliance (p = 0.017) (Loudon *et al*., 2009). Interestingly, when children felt safe and peer-relationships were secure, these secretive behaviours diminished and were instead replaced with children’s attempts to educate their peers (Koklanis, Abel and Aroni, 2006).

Multiple studies also demonstrated how poorer adherence to patching is associated with higher levels of parenting distress (Drews-Botsch *et al*., 2012; Drews-Botsch *et al*., 2019; Loudon *et al*., 2009; Searle *et al*., 2000). Parents with poorer compliance perceived that their children were less active, less engaged in physical activities, socialising, reading, and experienced difficulty when playing outdoors during treatment (Searle *et al*., 2000; Tjiam *et al*., 2011). The opposite was also demonstrated to be true; when parents experienced lower levels of stress, they reported much better compliance with patching (Drews-Botsch *et al*., 2012; Drews-Botsch *et al*., 2019; Kitasato *et al*., 2020).

### 3.5 Amblyogenic factor and severity

Levels of distress experienced by the patient due to amblyopia treatment appeared to be correlated with the underlying amblyogenic factor. Anisometropic amblyopes generally achieved lower stress level scores than those with strabismic and stimulus deprivation types of amblyopia, with some anisometropic amblyopes demonstrating similar levels of stress to those of non-amblyopic control groups (Guimarães *et al*., 2019). A similar effect has also been documented for social acceptance scores, with anisometropic amblyopes generating scores akin to those of age-matched control children, while individuals with strabismic amblyopia yielded significantly poorer scores than controls (p = 0.012) (Webber *et al*., 2008).

Amblyogenic factor was also associated with parental distress levels, with similar factor-patterns identified as those seen among amblyopic children. In their recent examination of perceived stress scales (PSS) in Saudi Arabian parents of amblyopic children, Alkulaib and colleagues (2021) concluded that relatively lower levels of distress were seen among the parents of children with anisometropic amblyopia than those with other amblyogenic factors. Additionally, they hypothesised that these lower perceived stress scores may be a reflection of better parental coping behaviours, or perhaps a more positive outlook associated with refractive/spectacle treatment (Alkulaib *et al*., 2021).

In consideration of severity of amblyopia, patients with greater amblyopic density (moderate and severe/dense amblyopia, 6/18 acuity and poorer) have been shown to experience greater treatment related distress than those with mild amblyopia (<6/12 acuity), presenting as higher proportions of moderate or strong objection to occlusion occurring in moderate and dense amblyopes, compared to mild amblyopes (64% vs 41% respectively) (Parkes, 2001). Additionally, significantly larger psychological impact scores (PIS) were recorded for teenagers with moderate or severe amblyopia (reflecting a larger negative affect of treatment) than those with mild amblyopia (p < 0.02) (Sabri *et al*., 2006). Finally, as the visual acuity threshold of the amblyopic eye improves and amblyopic density diminishes, Health Related Quality of Life (HRQoL) scores improve (Chen *et al*., 2016).

### 3.6 Treatment modality and duration

All treatment methods (refractive correction, patching occlusion, and atropine penalizing) were documented as exhibiting some level of distress. Evidence was seen for worries centred around refractive correction and patching occlusion (Choong *et al*., 2004; Dixon-Woods, Awan and Gottlob, 2006; Koklanis, Abel and Aroni, 2006), with both treatment modalities yielding moderate to high distress among Saudia Arabian parents of amblyopic children (Alkulaib *et al*., 2021). Greater concerns were seen regarding patching occlusion compared to refractive correction in both parent and patient groups (Hrisos, Clarke and Wright, 2004), with children developing more positive associations with their refractive corrections (such as considering themselves ‘smarter’ with their glasses) but not with their patch (Koklanis, Abel and Aroni, 2006). Conflicting results are seen regarding the longer-term impact of patching, with teenagers who had previously undertaken patching occlusion and defined it as an unpleasant experience yielding significantly higher (poorer) psychological impact scores (p < 0.0001, akin to those of patients with glaucoma) than those who found the experience acceptable (Sabri *et al*., 2006). However, no significant difference was seen in the psychosocial scores between refractive amblyopes (mean age of 12 years) who were treated with glasses alone, and those treated with glasses and patching who had ceased occlusion ≥1 year prior (p = 0.54 to p = 0.98) (Guimarães *et al*., 2019).

In consideration of atropine, the Amblyopia Treatment Index (ATI) distributed to families randomly assigned to either patching occlusion or atropine penalisation by the ‘Paediatric Eye Disease Investigator Group’ (Holmes *et al*., 2003) yielded higher (poorer) scores in the families who received patching occlusion versus those who received atropine penalisation (mean, 2.52 vs 2.02, p < 0.001). Further studies have replicated this finding with atropine penalisation demonstrating a diminished negative psychological impact compared to both patching occlusion (Felius *et al*., 2010) and even refractive correction (Koklanis, Abel and Aroni, 2006).

Regarding treatment duration, higher stress was seen in the parents/guardians who had administered amblyopia treatment for >4 years compared to those who had administered treatment for <1 year, although this difference did not reach statistically significance (93.5% vs. 82.6%, respectively; P = 0.561) (Alkulaib *et al*., 2021).

## 4. Discussion

The majority of studies contained in this systematic literature review consider only the guardian’s perspective of the patient or themselves (n = 16/25). While guardian perspectives are valuable, proxy reported outcome measures for paediatric populations are increasingly discouraged, in favour of patient self-perspective (FDA, 2009). Development of amblyopia specific patient-reported outcome (PRO) tools such as the Child Amblyopia Treatment Questionnaire (CAT-QoL, Carlton, 2013) and the Child Amblyopia Treatment Index (ATI, Felius *et al*., 2010), will help to readdress the balance between patient and guardian perspectives, regarding amblyopia treatment and its psychological impact.

While it is difficult to determine a clear consensus on the psychological impact of amblyopia treatment due to the low number of existing amblyopia specific questionnaires compared with the frequent use of other more generalised psychological assessments, and may limit the validity of these findings, this review has highlighted a range of conclusions regarding the psychosocial impact of amblyopia treatment upon both patients and their guardians, from no negative effect to significant negative psychological consequence. Interestingly, the amblyopia treatment experience is demonstrably memorable as every participant within the Sabri et al. (2006) study clearly remembered their amblyopia treatment experience, with no participant answering, ‘cannot remember’, regardless of whether they considered it unpleasant or not. For those whom amblyopia treatment affects negatively, such consequences included social isolation, ridicule, and bullying, reduced positive self-image, limitation of physical activities, as well as tension between the child, family members and teachers. While no ‘smoking gun’ is identified which assures negative psychological effects, predisposing factors such as presence of strabismus, more severe amblyopic density, occlusive patch treatment and patching during school age, appear to influence towards harsher psychosocial impact for both parents and children.

Despite frequent identification of treatment at school age as a factor for negative psychological effects such as distress, social isolation or diminished self-image, some studies found that most parents with school aged children (89–96%) believe that their children were coping well with their amblyopia treatment (Hrisos, Clarke and Wright, 2004), with ATI scores from the 2003 PEDIG study demonstrating that parents believed their 3–6-year-old children tolerated treatment well (Holmes *et al*., 2003). Therefore, there arises a conflict between the evidence supporting children’s tolerance throughout the amblyopia treatment process (Felius *et al*., 2010; Holmes *et al*., 2003; Hrisos, Clarke and Wright, 2004; Webber *et al*., 2008) and the evidenced long-term detrimental psychological impact of treatment (Packwood *et al*., 1999; Sabri *et al*., 2006). There is a lack of longitudinal research data to examine the correlation between a child’s behaviour and experiences during amblyopia treatment, with their adult psychological state, and so it is therefore possible that despite a child demonstrating a perceived tolerance for their amblyopia treatment at the time, they may still experience long-term detrimental psychological effects, particularly if they perceived the treatment process to be unpleasant. This possibility should be considered by both clinicians and parents during the treatment process, and further study conducted to examine and directly compare the immediate and long-term psychological states of amblyopic patients. Of note, in areas with no commissioned visual screening services, children may be at higher risk of later diagnosis and treatment of amblyopia, leading to increased negative psychological treatment effects associated with treatment of denser amblyopia and treatment at older ages.

Atropine penalisation appears to be the best tolerated treatment modality with the lowest psychosocial impact demonstrated; this is followed by refractive correction and finally patch occlusion. While atropine treatment has been shown to be equally as effective a treatment as an occlusive patch in cases of moderate amblyopia, it is frequently used as a secondary or alternative treatment method should compliance with patching occlusion be poor. Atropine also demonstrates less social stigma than occlusive patch wear, which is a primary concern for children especially once school age is reached. As visual screening is conducted in the UK between the ages of 4–5 years, the majority of amblyopia diagnoses will occur at approximately the beginning of school life. Therefore, offering atropine as a first line treatment should be considered as it offers greater psychosocial benefits over traditional patching occlusion, while providing equivalent treatment effectiveness for moderate amblyopia.

Additionally, while lower levels of distress are associated with greater parental compliance with patching treatment, the complete nature and direction of this relationship remains unclear. Psychological assessment both prior to, and post treatment commencement would be of value to establish the existence of correlation or causation in this matter.

The psychological impact of new amblyopia treatment developments, such as the use of dichoptic images to facilitate balanced binocular interaction, are as yet unresearched and therefore unknown. Future research should also look to examine the psychosocial effects of these newer treatments and compare them to more traditional methods.

Finally, clarifying whether amblyopia treatment is distressing for patients is important due to the physiological consequences of distress. An audiological study of the psychological impact of tinnitus in adults found increased levels of the stress hormone cortisol in hair, cortisol concentration (HCC) measurements, along with decreased levels of brain derived neurotrophic factor (BDNF) (Basso *et al*., 2022). Brain derived neurotrophic factor is considered to be a key modulator for ocular dominance plasticity (Kowiański *et al*., 2018; Mandolesi *et al*., 2005; Sasi *et al*., 2017), with monocularly deprived animal models demonstrating an imbalance of BDNF expression between the deprived eye (lower BDNF expression) and the non-deprived eye (Mandolesi *et al*., 2005). Via administration of intravitreal injection of exogenous BDNF to the deprived eye, or reduction of endogenous BDNF expression in the nondeprived eye, Mandolesi and colleagues (2005) were able to counteract the ocular dominance shift induced by monocular deprivation. It is therefore feasible that for those individuals who find the amblyopia treatment process distressing, may also face raised cortisol levels and decreased BDNF levels which in turn might hamper their responsiveness to amblyopia treatment by reducing neuroplasticity. To date, there have been no quantitative physiological measurements of distress (such as measurement of cortisol) in a treatment active amblyopic population. Future studies should aim to address this gap in the literature and look at the possible neurological consequences of such stress.

## 5. Conclusions

Both parents/guardians and patients can experience distress as a result of undertaking amblyopia treatment. The potential short- and long-term psychological impact of amblyopia treatment, and the factors which predispose towards more negative effects, should be taken into consideration by clinicians when creating a personalised treatment plan for their amblyopic patients, with clinicians aiming to ‘strike a balance’ between maintaining psychological wellbeing and successful amblyopia treatment. Modifiable factors such as amblyopia identification and treatment commencement at a younger age (which may in turn reduce amblyopic severity), and optional first-line atropine penalisation treatment, may help to improve treatment compliance and reduce any potential distress experienced. Further study into quantitative physiological measurement of distress in amblyopic patients and their guardians, such as measurement of hair cortisol concentration and BDNF, is advised to address gaps in the literature.

## Additional File

The additional file for this article can be found as follows:

10.22599/bioj.426.s1 Appendices. Appendix 1 to 4.
