# Supplementary material for: The Psychological Impact of Amblyopia Treatment: A Systematic Literature Review
Source: Br Ir Orthopt J. 2025 Jan 16;21(1):1–14. doi: 10.22599/bioj.426 (PMC11740718; doi:10.22599/bioj.426)
Supplement: Appendices. — Appendix 1 to 4. [file bioj-21-1-426-s1.pdf]

Appendix 1 – PRISMA 2020 checklist

Section and Topic	Item #	Checklist item	Location where item is reported
TITLE			
Title	1	Identify the report as a systematic literature review	Title
ABSTRACT			
Abstract	2	As per PRISMA abstracts checklist ([Microsoft Word - PRISMA 2009 Checklist.doc (prisma-statement.org)])	Abstract
INTRODUCTION			
Rationale	3	Describe the rationale for the review in the context of existing knowledge	1.0
Objectives	4	Provide an explicit statement of the objective(s) or question(s) the review addresses.	1.0
METHODS			
Eligibility criteria	5	Specify the inclusion and exclusion criteria for the review and how studies were grouped for the syntheses.	2.1
Information sources	6	Specify all databases, registers, websites, organisations, reference lists and other sources searched or consulted to identify studies. Specify the date when each source was last searched or consulted.	2.3
Search strategy	7	Present the full search strategies for all databases, registers and websites, including any filters and limits used.	2.3
Selection process	8	Specify the methods used to decide whether a study met the inclusion criteria of the review, including how many reviewers screened each record and each report retrieved, whether they worked independently, and if applicable, details of automation tools used in the process.	2.4
Data collection process	9	Specify the methods used to collect data from reports, including how many reviewers collected data from each report, whether they worked independently, any processes for obtaining or confirming data from study investigators, and if applicable, details of automation tools used in the process.	2.5
Data items	10a	List and define all outcomes for which data were sought. Specify whether all results that were compatible with each outcome domain in each study were sought (e.g. for all measures, time points, analyses), and if not, the methods used to decide which results to collect.	2.5
	10b	List and define all other variables for which data were sought (e.g. participant and intervention characteristics, funding sources). Describe any assumptions made about any missing or unclear information.	2.5
Study risk of bias	11	Specify the methods used to assess risk of bias in the included studies, including details of the tool(s) used, how	2.6

assessment		many reviewers assessed each study and whether they worked independently, and if applicable, details of automation tools used in the process.	
Effect measures	12	Specify for each outcome the effect measure(s) (e.g. risk ratio, mean difference) used in the synthesis or presentation of results.	2.7
Synthesis methods	13a	Describe the processes used to decide which studies were eligible for each synthesis (e.g. tabulating the study intervention characteristics and comparing against the planned groups for each synthesis (item #5)).	2.7
	13b	Describe any methods required to prepare the data for presentation or synthesis, such as handling of missing summary statistics, or data conversions.	2.7
	13c	Describe any methods used to tabulate or visually display results of individual studies and syntheses.	2.7
	13d	Describe any methods used to synthesize results and provide a rationale for the choice(s). If meta-analysis was performed, describe the model(s), method(s) to identify the presence and extent of statistical heterogeneity, and software package(s) used.	2.7
	13e	Describe any methods used to explore possible causes of heterogeneity among study results (e.g. subgroup analysis, meta-regression).	NA
	13f	Describe any sensitivity analyses conducted to assess robustness of the synthesized results.	NA
Reporting bias assessment	14	Describe any methods used to assess risk of bias due to missing results in a synthesis (arising from reporting biases).	2.6
Certainty assessment	15	Describe any methods used to assess certainty (or confidence) in the body of evidence for an outcome.	2.6
RESULTS			
Study selection	16a	Describe the results of the search and selection process, from the number of records identified in the search to the number of studies included in the review, ideally using a flow diagram.	3.0
	16b	Cite studies that might appear to meet the inclusion criteria, but which were excluded, and explain why they were excluded.	3.0
Study characteristics	17	Cite each included study and present its characteristics.	3.0
Risk of bias in studies	18	Present assessments of risk of bias for each included study.	3.0
Results of individual studies	19	For all outcomes, present, for each study: (a) summary statistics for each group (where appropriate) and (b) an effect estimate and its precision (e.g. confidence/credible interval), ideally using structured tables or plots.	3.0
Results of syntheses	20a	For each synthesis, briefly summarise the characteristics and risk of bias among contributing studies.	3.0
	20b	Present results of all statistical syntheses conducted. If meta-analysis was done, present for each the summary	NA

		estimate and its precision (e.g. confidence/credible interval) and measures of statistical heterogeneity. If comparing groups, describe the direction of the effect.	
	20c	Present results of all investigations of possible causes of heterogeneity among study results.	NA
	20d	Present results of all sensitivity analyses conducted to assess the robustness of the synthesized results.	NA
Reporting biases	21	Present assessments of risk of bias due to missing results (arising from reporting biases) for each synthesis assessed.	2.6
Certainty of evidence	22	Present assessments of certainty (or confidence) in the body of evidence for each outcome assessed.	2.6
DISCUSSION			
Discussion	23a	Provide a general interpretation of the results in the context of other evidence.	4.0
	23b	Discuss any limitations of the evidence included in the review.	4.0
	23c	Discuss any limitations of the review processes used.	4.0
	23d	Discuss implications of the results for practice, policy, and future research.	4.0
OTHER INFORMATION			
Registration and protocol	24a	Provide registration information for the review, including register name and registration number, or state that the review was not registered.	2.0
	24b	Indicate where the review protocol can be accessed, or state that a protocol was not prepared.	2.0
	24c	Describe and explain any amendments to information provided at registration or in the protocol.	NA
Support	25	Describe sources of financial or non-financial support for the review, and the role of the funders or sponsors in the review.	7.0
Competing interests	26	Declare any competing interests of review authors.	8.0
Availability of data, code and other materials	27	Report which of the following are publicly available and where they can be found: template data collection forms; data extracted from included studies; data used for all analyses; analytic code; any other materials used in the review.	NA

2

Appendix 2 – Search databases and Terms

Databases:

- MEDLINE (1950 – June 2023)
- EMBASE (1980 - June 2023)
- CINAHL (1982 – June 2023)
- PsychINFO (1967 – Jun 2023)
- Web of Science
- Scopus

Search terms:

Amblyopia/	Psychological distress/
lazy eye	Distress, Psychological/
occlusion therapy	Emotional distress/
amblyopia treatment	Distress, Emotional/
	Emotional Stress/
	Stress, Emotional/
	stress
	distress
	worry
OR	OR
AND	

Appendix 3 – Adapted Newcastle-Ottowa score

Adapted Version of the Newcastle-Ottawa Scale Used in the Present Study for Quality Assessment (Maximum 7 Stars). Reproduced from Tramontano et al, 2021.

Selection: (maximum 2 stars)

1) Representativeness of the general population sample:

   a) Truly representative of the average in the target population.* (all subjects or random sampling)

   b) Somewhat representative of the average in the target population.* (non-random sampling)

   c) Selected group of users.

   d) No description of the sampling strategy.

2) Sample size:

   a) Satisfactory.* (MIN 30)

   b) Not satisfactory.

Assessment/Treatment protocol: (maximum 2 stars)

a) The treatment protocol is described.**

b) No description of the treatment protocol.

Outcome(s): (maximum 3 stars)

1) Assessment of the outcome:

   a) Detailed description.**

   b) No description.

2) Statistical test:

   a) The statistical test used to analyse the data is clearly described and appropriate, and the measurement of the association is presented, including confidence intervals and the probability level (*P* value).*

   b) The statistical test is not appropriate, not described, or incomplete.

Adapted NOS Scores

	1) Representativeness of the general population sample	2) Sample size	3) Assessment/ Treatment protocol	3) Assessment of the outcome	4) Statistical test	TOTAL STARS
**Alkulaib (2021)**	*	*	**	**	*	7
**Bhandari (2012)**	*	*	**	**	*	7
**Carlton (2013)**	*	*	**	**		6
**Chen (2016)**	*	*	**	**	*	7
**Choong (2004)**	*	*	**	**	*	7
**Cole (2001)**	*	*	**	**	*	7
**Dixon-Woods (2006)**	*		**	**		5
**Drews (2003)**	*	*	**	**	*	7
**Drews-Botsch (2012)**	*	*	**	**	*	7
**Drews-Botsch (2019)**	*	*	**	**	*	6
**Felius (2010)**	*	*	**	**	*	7
**Guimares (2019)**		*	**	**	*	6
**Holmes (2003)**	*	*	**	**	*	7
**Holmes (2008)**	*	*	**	**	*	7
**Hrisos (2004)**	*	*	**	**	*	7
**Kitatso (2020)**	*	*	**	**		6
**Koklanis (2006)**	*	*	**	**	*	6
**Loudon (2009)**	*	*	**	**	*	7
**Packwood (1999)**	*		**	**	*	6
**Parkes (2001)**	*	*	**	**	*	7
**Sabri (2006)**	*	*	**	**	*	7
**Searle (2000)**			**	**		4
**Searle (2002)**	*	*	**	**		6
**Tjiam (2011)**		*	**	**	*	6
**Webber (2008)**	*	*	**	**	*	7

Appendix 4 – Details of the ATI (Cole *et al.,* 2001; Holmes *et al.,* 2003; Holmes *et al.,* 2008; Felius *et al.,* 2010) and the Child Amblyopia Treatment Questionnaire (CAT-QoL, Carlton, 2013).

Parent and child amblyopia treatment index items:

Atropine treatment version (replicated from Felius *et al.,* 2010)

Parent ATI						Child ATI					
Strongly agree (5)	Agree (4)	Neither agree nor disagree (3)	Disagree (2)	Strongly Disagree (1)	NA	Strongly agree (5)	Agree (4)	Neither agree nor disagree (3)	Disagree (2)	Strongly Disagree (1)	NA
No.	Item					No.	Item				
1	My child does not seem to mind using the drops					1	It bothers me to use the drops				
2	I worry that by using the drops, my child may miss out on fun activities (such as games, sports, and parties).					2	I can't do fun things (such as games and sports) because of the drops.				
3	Using the drops affects my child's learning.					3	The drops make my schoolwork harder				
4	Using the drops makes it hard for my child to play outside, such as riding a bike.					4	The drops make it hard for me to play outside.				
5	I have trouble putting the drops in my child's eye.					5	It's hard to get drops put in my eye.				
6a	Using the drops is a source of tension or conflict in my relationship with my child.										
6b	Using the drops is a source of tension or conflict in my relationship with another family member					6	The drops make my parents argue				

6c	Using the drops is a source of tension or conflict in my relationship with my child's teacher	7	The drops make others in my family argue.
7	Using the drops makes it difficult for my child to read or write.	8	The drops make it hard to read and write
8	I worry that my child will become injured when using the drops	9	I worry that I will run into things because of the drops
9	My child can see well when using the drops	10	I can see well when the drops are in
10	My child complains when it is time to put in the drops.	11	I don't like it when it's time for the drops
11	Using the drops makes my child's eye or eyelids red or irritated	12	The drops make my eyes or eyelids red
12	I worry that my child does not get the drops often enough	13	I worry that I don't get enough drops
13	My child is more clumsy and uncoordinated than usual when using the drops	14	The drops make me clumsy
14	I notice that other children stare at my child when the drops are in	15	My friends stare at my eye when the drops are in
15	I believe that using the drops will improve my child's vision	16	I think the drops will help me see better
16	Using the drops makes it difficult for my child to play with small toys or hand-held video games	17	The drops make it hard to play with small toys or hand-held video games
17	I sometimes forget to put the drops in my child's eye	18	My parents forget to put the drops in
18	I worry that using the drops will make my child feel different from other children.	19	The drops make me feel different from my friends.

Child Amblyopia Treatment Questionnaire (CAT-QoL, replicated from Carlton, 2013).

11 themes were identified during qualitative analysis of interview data for possible inclusion in the CAT-QoL draft questionnaire, these included:

1. Physical sensation of the treatment (e.g., feeling of the patch/glasses on the face or the feeling of the drops being instilled)
2. Pain of treatment (hurt)
3. Being able to play with other children
4. How other children have treated them (like laughing or name calling)
5. Ability to undertake work at school
6. Ability to undertake other tasks (like playing on the computer, colouring, playing games, watching TV)
7. Feeling sad or unhappy
8. Feeling cross
9. Feeling worried
10. Feeling frustrated
11. Feelings toward family members (like parents or siblings)

These themes were worked into an 11-point draft questionnaire which was piloted are refined into refined a CAT-QoL instrument that consists of 8-items, each with three-level response scales (Carlton, 2019).

Eight item CAT-QoL (Patch version, replicated from Carlton, 2019)

**Item**		Score
**Sad**	My patch has not made me feel sad	0
	My patch has made me feel a little bit sad	1
	My patch has made me feel very sad	2
**Feeling of your patch on your face (like sticky, or itchy)**	The feel of my patch has not bothered me	0
	The feel of my patch has bothered me a bit	1
	The feel of my patch has bothered me a lot	2
**Hurt**	My patch did not hurt me	0
	My patch hurt me a bit	1
	My patch hurt me a lot	2
**Doing work at school (like reading or writing)**	My patch has not made it hard to do my work	0
	My patch made it a bit hard to do my work	1
	My patch made it very hard to do my work	2
**How other children have treated you (like laughing at you, or calling you names), because of your patch**	Children have not laughed at me or called me names	0
	Children have laughed at me or called me names a bit	1
	Children have laughed at me or called me names a lot	2
**Doing things (like playing on the computer, colouring, playing games, watching TV)**	My patch has not made it hard to do things	0
	My patch has made it a bit hard to do things	1
	My patch has made it very hard to do things	2
**Worried**	My patch has not made me feel worried	0
	My patch has made me feel a bit worried	1
	My patch has made me feel very worried	2
**Playing with my friends**	My patch has not stopped me playing with my friends	0
	My patch has stopped me playing with my friends a bit	1
	My patch has stopped me playing with my friends a lot	2
